# Necrotizing fasciitis after high-dose rate brachytherapy and external beam radiation for prostate cancer: a case report

**DOI:** 10.1186/s12894-017-0299-y

**Published:** 2017-11-21

**Authors:** Shimpei Yamashita, Yasuo Kohjimoto, Akinori Iba, Kazuro Kikkawa, Isao Hara

**Affiliations:** Department of Urology, Wakayama Medical University, 811-1 Kimiidera, Wakayama, 641-8509 Japan

**Keywords:** Prostate cancer, High-dose rate brachytherapy, Necrotizing fasciitis, Intra-wound continuous negative pressure and irrigation treatment

## Abstract

**Background:**

In recent years, the delayed side effects associated with radiotherapy for prostate cancer have drawn the interest of urologists. Although urosymphyseal fistula is one of these delayed side effects, this serious complication is rarely described in literature and is poorly recognized.

**Case presentation:**

We report our experience in treating a 77-year-old male patient with necrotizing fasciitis after high-dose rate brachytherapy plus external beam radiation for prostate cancer. The patient was referred to our hospital with complaints of inguinal swelling and fever. He had a past history of radiotherapy for prostate cancer and subsequent transurethral operation for a stricture of the urethra. Computed tomography showed extensive gas within the femoral and retroperitoneal tissues and pubic bone fracture. Surgical exploration suggested that necrotizing fasciitis was caused by urosymphyseal fistula.

**Conclusion:**

To the best of our knowledge, this is the first case report of necrotizing fasciitis caused by urosymphyseal fistula after radiotherapy for prostate cancer. There is a strong association between urosymphyseal fistula and prostate radiotherapy with subsequent surgical intervention for bladder neck contracture or urethral stricture. Therefore, surgical treatment for bladder neck contracture or urethral stricture after radiotherapy for prostate cancer should be performed with care.

The present case emphasizes the importance of early diagnosis of urosymphyseal fistula. Immediate removal of necrotic tissues and subsequent urinary diversion in the present case may have led to good patient outcome.

## Background

Radiotherapy for prostate cancer is generally regarded as a less invasive treatment than prostatectomy. On the other hand, patients who undergo radiotherapy have higher incidence of complications requiring hospital admissions, rectal or anal procedures, open surgical procedures, and secondary malignancies at five years compared with those who undergo surgery [1]. However, information on delayed side effects associated with radiotherapy for prostate cancer remains limited. Although urosymphyseal fistula is one of these delayed side effects [2, 3], this serious complication is rarely described in literature and is poorly recognized.

We report a case of femoral necrotizing fasciitis caused by puboprostatic fistula after high-dose rate brachytherapy plus external beam radiation for prostate cancer.

## Case presentation

A 77-year-old male patient was diagnosed with prostate cancer on biopsies taken because of a high prostate specific antigen (PSA) serum concentration (10.35 ng/mL). Histological examination revealed prostate cancer with Gleason score 6 (3 + 3) and the clinical stage was determined as T1cN0M0 by magnetic resonance imaging (MRI), computed tomography (CT) and bone scintigraphy. The patient had a previous history of trans-urethral resection of prostate (TURP) for benign prostatic hyperplasia more than ten years prior. He was not suffering from diabetes mellitus, hypertension or neurogenic bladder. He was administered with external beam radiation at a total dose of 50 Gy in 25 fractions and high-dose rate brachytherapy at a total dose of 18 Gy in 2 fractions. After treatment, serum PSA value immediately decreased (nadir value 0.21 ng/mL).

**Fig. 1:**
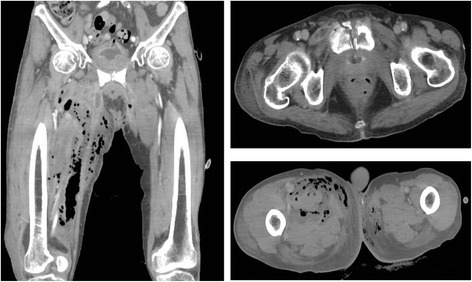
CT scan showed extensive gas within the femoral and retroperitoneal tissues and pubic bone fracture

Three years later, the patient received medical examination due to dysuria during follow up. Cystoscopy revealed that prostatic urethra was obstructed by yellow necrotic tissue, and prostatic and membranous urethral mucous membrane were white and hemorrhagic. These abnormal findings were thought to be caused by previous radiation therapies. As urethral catheterization was difficult because of the obstruction, he received transurethral resection of the necrotic tissue. Resected tissue did not contain viable cancer. However, after the operation, complete urinary incontinence occurred and gross hematuria and pyuria persisted. Several months later, he returned to the hospital due to inguinal swelling and fever. His inguinal and femoral region turned purple with pus present. Leukocytosis was observed (white cell count 12,870 /mm^3^), and CRP was elevated at 22.28 mg/dL. CT scan showed extensive gas within the femoral and retroperitoneal tissues and pubic bone fracture (Fig. 1). He underwent immediate surgical exploration for suspected necrotizing fasciitis. Exploration of both the groin and right thigh revealed severe soft tissue infection involving the skin, subcutaneous fat and fascia. The pubic bone became necrotic and the ventral part of the prostate was absent, thereby exposing the urethral catheter. These operative findings suggested necrotizing fasciitis was caused by urinary fistulation into the pubic symphysis and subsequent pubic symphysis osteomyelitis. Microbiological exam showed growth *of E. coli* and *Enterococcus faecalis* and patient was treated with broad-spectrum antibiotics. Simultaneously, negative pressure wound therapy was performed. The patient underwent ureterocutaneous urinary diversion on day 8 after surgery. On day 148 post surgery, the patient’s wound recovered and he regained mobility.

## Discussion

In general, patients who undergo radiotherapy are thought to have lower incidence of urinary incontinence and erectile dysfunction than those who undergo prostatectomy. However, a recent study with long-term follow-up identified no significant relative differences in disease-specific functional outcomes, such as urinary incontinence and erectile dysfunction, among men who underwent prostatectomy or radiotherapy [4]. Furthermore, patients who were given radiotherapy had higher incidence of severe complications at five years than those who underwent surgery [1]. However, information about the late side effects associated with radiotherapy for prostate cancer remains limited.

We reported a case of femoral necrotizing fasciitis caused by puboprostatic fistula after transurethral resection for urethral stricture subsequent to radiotherapy for prostate cancer. The patient course provided an important clinical suggestion; transurethral treatment for stricture of the urethra after radiotherapy for the prostate requires caution because of potential urosymphyseal fistula (USF) can lead to serious infection, such as necrotizing fasciitis.

Recently, USF has been reported to be a serious complication after treatment of prostate cancer [2, 3]. Matsushita et al. reported 12 cases of pubovesical fistula after treatment of prostate cancer, and all cases underwent radiotherapy (as primary treatment for prostate cancer in eight cases and as salvage treatment in four cases) and subsequent endoscopic treatment for bladder neck contracture (BNC) [3]. Bugeja et al. described 16 cases of urosymphyseal fistula after of prostate cancer. All patients underwent radiotherapy (as primary treatment in eight cases and as salvage or adjuvant treatment in eight cases) and 13 of the 16 patients (81.3%) underwent some form of corrective surgical intervention (open surgical revision of BNC in four cases, bladder neck incision in two cases, dilation of BNC in two cases and TURP in five cases). These studies have suggested a strong association between USF and prostate radiotherapy with subsequent surgical intervention for BNC or urethral stricture. Also in the present case, we performed transurethral resection for urethral stricture after radiotherapy for prostate cancer. We hypothesized that insufficient regeneration of urinary tract tissue associated with radiotherapy was responsible for pubosymphyseal fistula and subsequent necrotizing fasciitis. Although some above studies reported USF after treatment for prostate cancer, we think that this severe complication remains poorly recognized. Therefore, it is necessary to keep in mind that surgical treatment for BNC or urethral stricture after radiotherapy for prostate cancer should be performed carefully because of potential subsequent USF.

To our knowledge, this is the first case report of necrotizing fasciitis caused by USF after radiotherapy for prostate cancer. Fournier’s gangrene is a representative necrotizing fasciitis in the urological field. However, in this case, infection spread through a specific route; from cavity of the pelvis to the thighs through an obturator foramen. The present case emphasizes the importance of early diagnosis of USF. Previous studies have reported that typical symptoms of USF are recurrent urinary tract infection and pelvic pain, and MRI is useful for diagnosis of this disease as well as CT and cystoscopy [2, 3]. Although conservative treatment with antibiotics and temporary urinary diversion by suprapubic tube replacement or bilateral nephrostomy tube placement are usually the first attempts, almost all patients subsequently need cystectomy with permanent urinary diversion [2, 3]. In our case, we gave priority to saving the patient’s life and performed only urinary diversion to stop urinary leakage together with the treatment of femoral necrotizing fasciitis. Necrotizing fasciitis is a rapidly progressive infection of soft tissues and a potentially life-threatening disease and mortality rates were estimated between 25 and 35% [5]. We believe that timely urinary diversion was a key factor in the treatment of this patient.

## Conclusions

In conclusion, surgical treatment for BNC or urethral stricture after radiotherapy for prostate cancer should be performed carefully because of potential subsequent USF. If BNC or stricture of the urethra occur, we need to carefully consider the indication of the transurethral operation and the option to provide an early urinary diversion. A large-scale study is warranted to investigate the long-term progress after radiotherapy for prostate cancer.

## Abbreviations

BNC: Bladder neck contracture
CRP: C-reactive protein
CT: Computed tomography
MRI: Magnetic resonance imaging
PSA: Prostate specific antigen
TURP: Trans-urethral resection of prostate
USF: Urosymphyseal fistula
